# Myeloid-derived suppressor cells regulate the immunosuppressive functions of PD-1^−^PD-L1^+^ Bregs through PD-L1/PI3K/AKT/NF-κB axis in breast cancer

**DOI:** 10.1038/s41419-021-03745-1

**Published:** 2021-05-09

**Authors:** Min Liu, Feng Wei, Jian Wang, Wenwen Yu, Meng Shen, Ting Liu, Dong Zhang, Yang Wang, Xiubao Ren, Qian Sun

**Affiliations:** Department of Immunology, Tianjin Medical University Cancer Institute and Hospital, National Clinical Research Center for Cancer, Key Laboratory of Cancer Prevention and Therapy, Key Laboratory of Cancer Immunology and Biotherapy, Tianjin’s Clinical Research Center for Cancer, Tianjin, 300060 China

**Keywords:** Breast cancer, Cancer microenvironment

## Abstract

Myeloid-derived suppressor cells (MDSCs) are a heterogeneous group of myeloid cells that are closely related to tumor immune escape, but the mechanism by which MDSCs regulate B cells has not been elucidated. Our previous studies revealed that breast cancer-derived MDSCs could induce a group of PD-1^−^PD-L1^+^ Bregs with immunosuppressive functions. Here, we reported that blocking PD-1/PD-L1 interaction between MDSCs and B cells could reverse the immunosuppressive functions of PD-1^−^PD-L1^+^ Bregs. The activation of PI3K/AKT/NF-κB signaling pathway is essential for PD-1^−^PD-L1^+^ Bregs to exert immunosuppressive effects. MDSCs activated the PI3K/AKT/NF-κB pathway in B cells via the PD-1/PD-L1 axis. Furthermore, inhibition of PD-1/PD-L1 or PI3K/AKT signaling suppressed both tumor growth and the immunosuppressive functions of PD-1^−^PD-L1^+^ Bregs. Dual suppression of PD-1/PD-L1 and PI3K/AKT exerted better antitumor effect. Finally, MDSCs and PD-1^−^PD-L1^+^ Bregs were colocalized in breast cancer tissues and PD-1^−^PD-L1^+^ Bregs were positively correlated with poor prognosis. Thus, MDSC-educated PD-1^−^PD-L1^+^ Bregs and their regulatory mechanisms could contribute to the immunosuppressive tumor microenvironment. Our study proposes a novel mechanism for MDSC-mediated regulation of B cell immunity, which might shed new light on tumor immunotherapy.^+^

## Introduction

Myeloid-derived suppressor cells (MDSCs) are a heterogeneous population of macrophages, dendritic cells, and granulocyte precursor or progenitor cells that negatively regulate the immune functions of tumor-bearing hosts and are considered to be one of key immunosuppressive cell types in the tumor microenvironment^1,2^. MDSCs mainly exert immunosuppressive effects by producing active substances such as arginase^3^, reactive oxygen species^4^, and nitric oxide^5^. Other studies have shown that MDSCs can downregulate the expression of CD62L, a molecule that is necessary for naive T cell homing, which prevents naive T cells from effectively migrating to lymph nodes (LNs), where they can be stimulated by tumor antigens, resulting in a reduction in the number of activated CD4^+^ and CD8^+^ T cells^6^. MDSCs can also induce the production of regulatory T cells by secreting interleukin-10 and transforming growth factor-β, thereby indirectly affecting the activation of T cells^7,8^. In addition, MDSCs were found to exert their immunosuppressive effects by regulating macrophages, dendritic cells, and natural killer cells^9,10^. Therefore, it is of great importance to study the immunosuppressive mechanism of MDSCs in antitumor immunotherapy^11,12^.^+^^+^

As one of the critical components of the adaptive immune response, B cells have also been shown to be important in the field of tumor immunity^13^. B cells not only mediate humoral immunity but also present tumor antigens to T cells as antigen-presenting cells^14^. Notably, the appearance and importance of regulatory B cells (Bregs) with immunosuppressive functions are associated with different immune-related pathologies^15^. For this reason, an increasing number of studies have better characterized the signals that induce the differentiation of Bregs to exploit their therapeutic potential^16,17^.

The tumor microenvironment is composed of a variety of immune cells and tumor stromal cells and is a very immunosuppressed milieu^18^. The causes and mechanisms of immunosuppression are also very complex, and immunosuppressive cells are one component^19^. Previous studies^20,21^ have shown that MDSCs have a rich and thorough history of regulating the responses of immune cells, such as T cells and natural killer cells. However, the effects of MDSCs on the function of B cells have not been fully elucidated. In autoimmune diseases, MDSCs can induce B cell expansion by inducible nitric oxide synthase^22^ or regulate B cell function by inhibiting autologous B cell proliferation and antibody (Ab) production^23^. Additionally, MDSCs control the accumulation and cytokine secretion of B cells in the inflamed central nervous system^24^. However, these findings have not fully elucidated the interactions of MDSCs with B cells, especially in tumors.

We previously reported that tumor-derived MDSCs could educate a unique Breg subpopulations in vitro, which defined as programmed death-1-negative, programmed death ligand 1-positive (PD-1^−^PD-L1^+^) B cell that exhibits a stronger inhibitory effect on T cell immune responses than traditionally defined Bregs (CD5^+^ B cells)^25^. Our present study was designed to elaborate the regulatory mechanism of MDSCs on PD-1^−^PD-L1^+^ Bregs. We demonstrated that tumor-derived MDSCs activated the phosphatidylinositol 3-kinase (PI3K)/protein kinase B (AKT)/nuclear factor kappa B (NF-κB) signaling pathway in B cells through the PD-1/PD-L1 axis, which affected the immunosuppressive functions of PD-1^+^PD-L1^+^ B cells (evaluated by T cell proliferation and interferon (IFN)-γ secretion). Clinical specimens were further examined and the results revealed that PD-1^−^PD-L1^+^ Bregs was associated with poor prognosis of breast cancer patients. In summary, the present study proposes a new way by which MDSCs mediate immune escape by enhancing the immunosuppressive functions of B cells in cancer, which provides more experimental evidence for exploring the applications of tumor immunotherapy.^+^

## Results

### Blocking PD-1/PD-L1 interaction reverses the immunosuppressive effects of MDSC-educated B cells

**Fig. 1:**
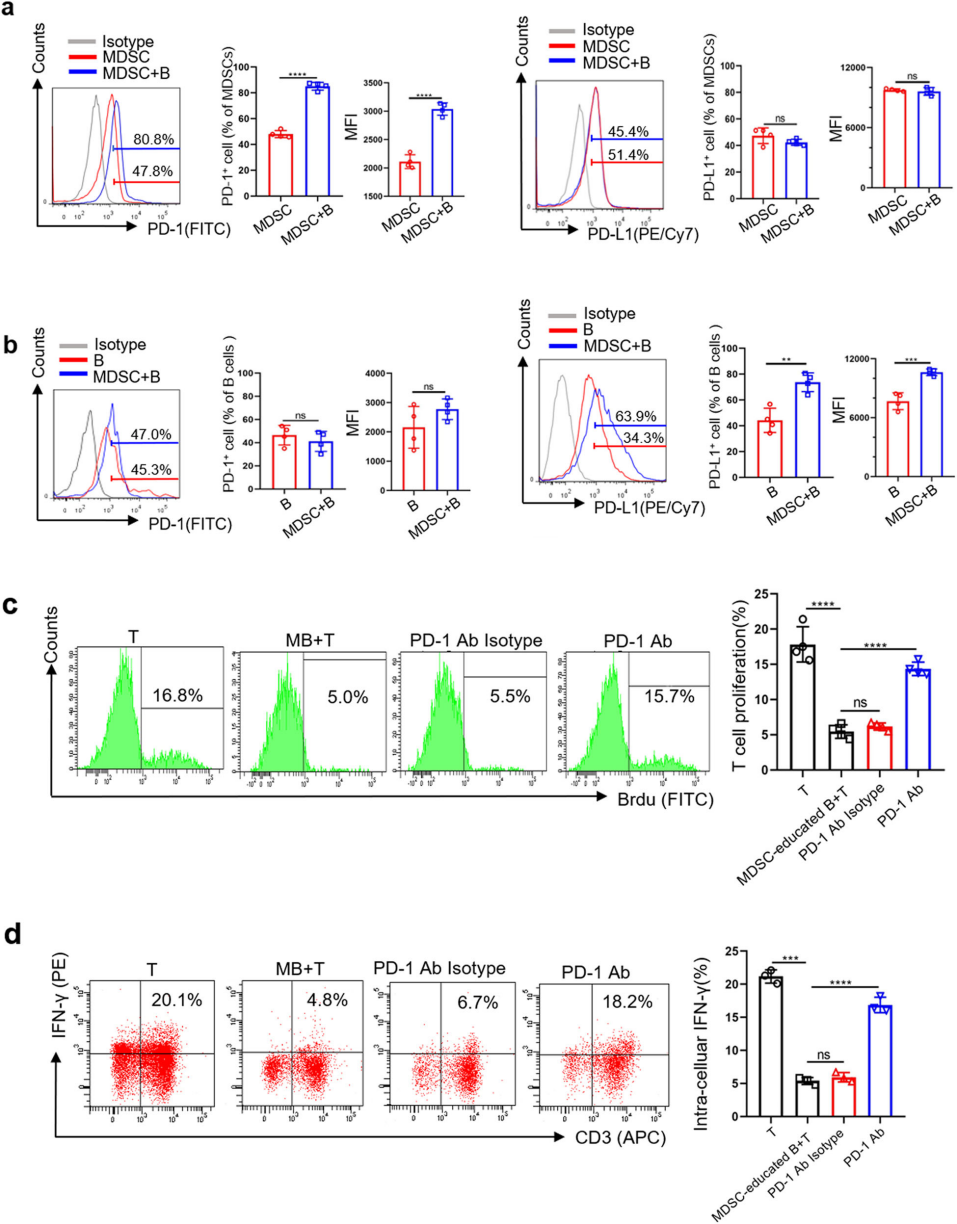
Blocking PD-1/PD-L1 interaction reverses the immunosuppressive effects of MDSC-educated B cells. **a** PD-1 and PD-L1 expression on MDSCs alone or cocultured with B cells (*n* = 4). **b** PD-1 and PD-L1 expression on B cells alone or cocultured with MDSCs (*n* = 4). **c**, **d** MDSCs were cocultured with B cells in the presence or absence of Ultra-LEAF™ Purified anti-mouse PD-1 Ab (20 µg/ml, Biolegend, USA) and isotype Ab (20 µg/ml, Biolegend, USA). After 24 h, these MDSC-educated B cells were separated and cocultured with T cells for 48 h. T cell proliferation and IFN-γ secretion were analyzed by FC (*n* = 4). Mean fluorescence intensity, MFI. (In all experiments, Bar graphs and plots represent or include mean ± SD, respectively. ns no statistically significant, *p < 0.05, **p < 0.01, ***p < 0.001, ****p < 0.0001).

To determine the role of the PD-1/PD-L1 axis in MDSCs and B cells, we first detected the expression levels of PD-1 and PD-L1 on MDSCs and B cells in coincubation system. The expression of PD-1 on MDSCs (Fig. 1a) and the expression of PD-L1 on B cells (Fig. 1b) in coincubation system were significantly increased compared to the control group, indicating that PD-1/PD-L1 axis may play an important role in MDSC regulation on B cells. We further added Ultra-LEAF™ purified anti-mouse PD-1 Abs to block the biological interaction of PD-1 and PD-L1 in the coincubation system. After 24 h, MDSC-educated B cells were isolated by magnetic cell sorting and then co-incubated with normal T cells to further evaluate its immunosuppressive function. We found that the inhibition of T cell proliferation (Fig. 1c) and IFN-γ secretion (Fig. 1d) by MDSC-educated B cells was reversed in the anti-PD-1 Ab groups, which suggests that MDSCs regulate the immunosuppressive functions of B cells through the PD-1/PD-L1 axis. Considering PD-L1 was highly expressed on MDSC-educated B cells, we speculate that the binding of PD-1 on MDSCs and PD-L1 on B cells will lead to MDSC-educated Bregs to exert immunosuppressive effects on T cell immune response.

### The activation of PI3K/AKT/NF-κB signaling pathway is essential for PD-1^−^PD-L1^+^ Bregs to exert immunosuppressive function^+^

**Fig. 2:**
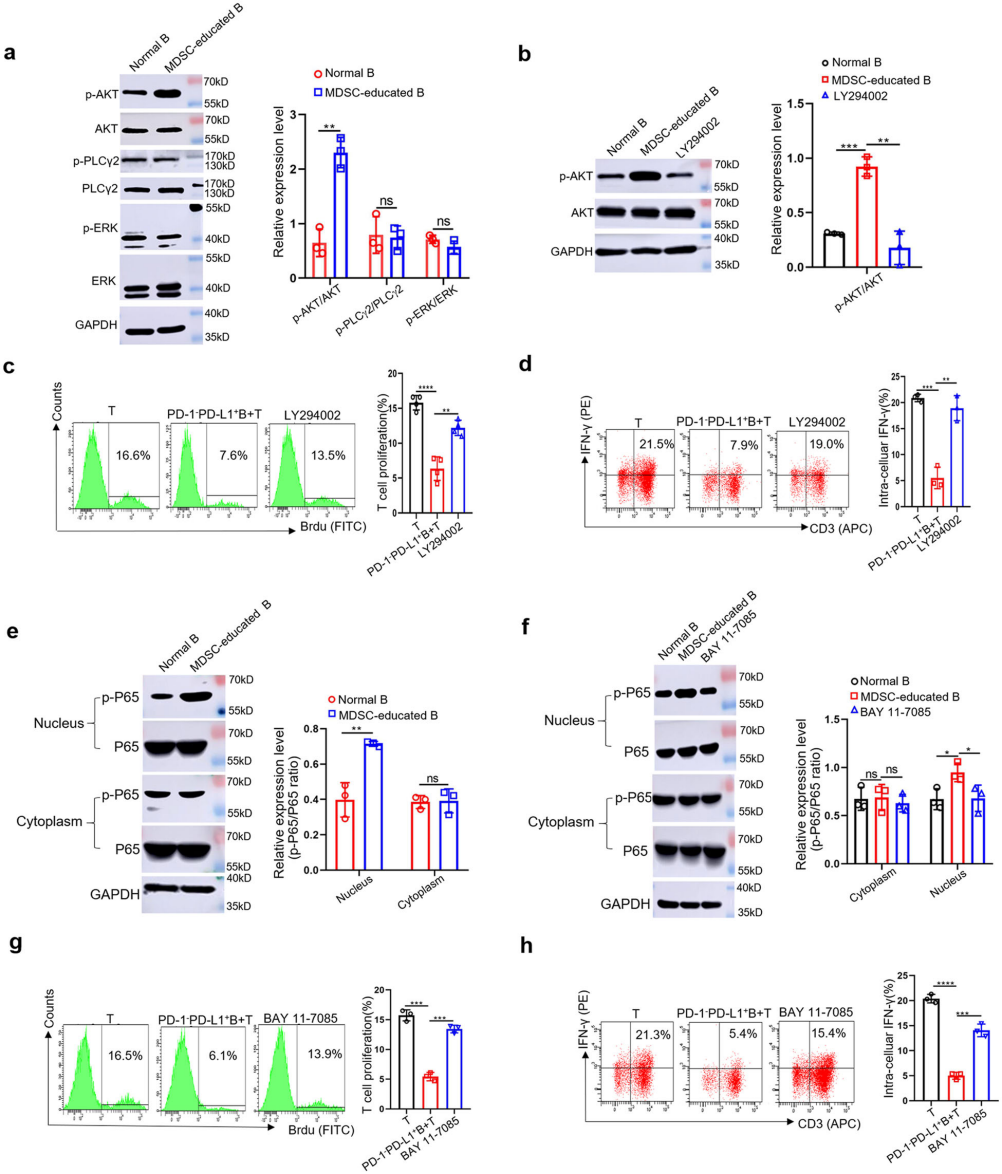
The activation of PI3K/AKT/NF-κB signaling pathway is essential for PD-1^−^PD-L1^+^ Bregs to exert immunosuppressive function. **a** The protein levels of AKT, p-AKT, PLCγ2, p-PLCγ2, ERK, and p-ERK in normal B cells and MDSC-educated B cells were measured by western blotting (*n* = 3). **b** Normal B and MDSC-educated B cells were treated with or without LY294002 (5 μM), the protein levels of AKT and p-AKT were detected (*n* = 3). **c**, **d** PD-1^−^PD-L1^+^ Bregs were treated with or without LY294002 (5 μM) and cocultured with normal T cells for 48 h. T cell proliferation and IFN-γ secretion were analyzed by FC (*n* = 4). **e** The protein levels of P65 and p-P65 in normal B cells and MDSC-educated B cells were measured by western blotting (*n* = 3). **f** Normal B and MDSC-educated B cells were treated with or without BAY 11-7085 (10 μM). The cytoplasmic and nuclear protein levels of P65 and p-P65 were measured by western blot (*n* = 3). **g**, **h** PD-1^−^PD-L1^+^ Bregs were treated with or without BAY 11-7085 (10 μM) and cocultured with normal T cells for 48 h. T cell proliferation and IFN-γ secretion were analyzed by FC (*n* = 3). (In all experiments, Bar graphs and plots represent or include mean ± SD, respectively. ns no statistically significant, *p < 0.05, **p < 0.01, ***p < 0.001, ****p < 0.0001).

Previous reports revealed that phospholipase C (PLC)γ2, extracellular signal-related kinase (ERK), and AKT were related to PD-1/PD-L1 signaling in B cells^26^. Thus, we evaluated these pathways in MDSC-educated PD-1^−^PD-L1^+^ Bregs. As the number of PD-1^−^PD-L1^+^ Bregs accounts for 60–70% of total MDSC-educated B cells^25^, we used the total population of MDSC-educated B cells instead of PD-1^−^PD-L1^+^ Bregs to measure protein levels. We found that the phosphorylation level of AKT in MDSC-educated B cells was significantly increased compared with that of normal B cells (Fig. 2a), while there was no significant difference in the phosphorylation level of PLCγ2 or ERK (Fig. 2a). We next examined whether AKT signaling is involved in the immunosuppressive effects of PD-1^−^PD-L1^+^ Bregs. Different concentration of LY294002 (an AKT signaling inhibitor) was assessed and we found that 5 µM is the optimum concentration for B cells to maintain its proliferation activity (Supplementary Fig. S1a). After treatment with 5 µM LY294002 (Fig. 2b), PD-1^−^PD-L1^+^ Bregs were collected and incubated with T cells to detect T cell proliferation and IFN-γ secretion by flow cytometry (FC). The results showed that PD-1^−^PD-L1^+^ Bregs no longer inhibited T cell proliferation (Fig. 2c) or IFN-γ secretion (Fig. 2d) after treatment with LY294002, suggesting that the PI3K/AKT signaling pathway is crucial for the immunosuppressive effects of PD-1^−^PD-L1^+^ Bregs.

NF-κB are the main effector molecules of PI3K/AKT downstream signaling^27–29^. We further examined the phosphorylation of P65 (marker of NF-κB activation)^30^ in MDSC-educated B cells. Significant upregulation of P65 phosphorylation was observed in MDSC-educated B cells compared with that of normal B cells (Fig. 2e). Then we used appropriate concentration of BAY 11-7085 (an NF-κB inhibitor) to reduce the P65 activation in PD-1^−^PD-L1^+^ Bregs (Supplementary Fig. S1b and Fig. 2f). As expected, treatment with BAY 11-7085 partially reversed PD-1^−^PD-L1^+^ Bregs-mediated inhibition of T cell proliferation (Fig. 2g) and IFN-γ secretion (Fig. 2h). These data suggest that the PI3K/AKT/NF-κB signaling pathways play important roles in the immunosuppressive effects of PD-1^−^PD-L1^+^ Bregs.

### Tumor-derived MDSCs activate the PI3K/AKT/NF-κB signaling pathway in B cells through PD-1/PD-L1 axis

**Fig. 3:**
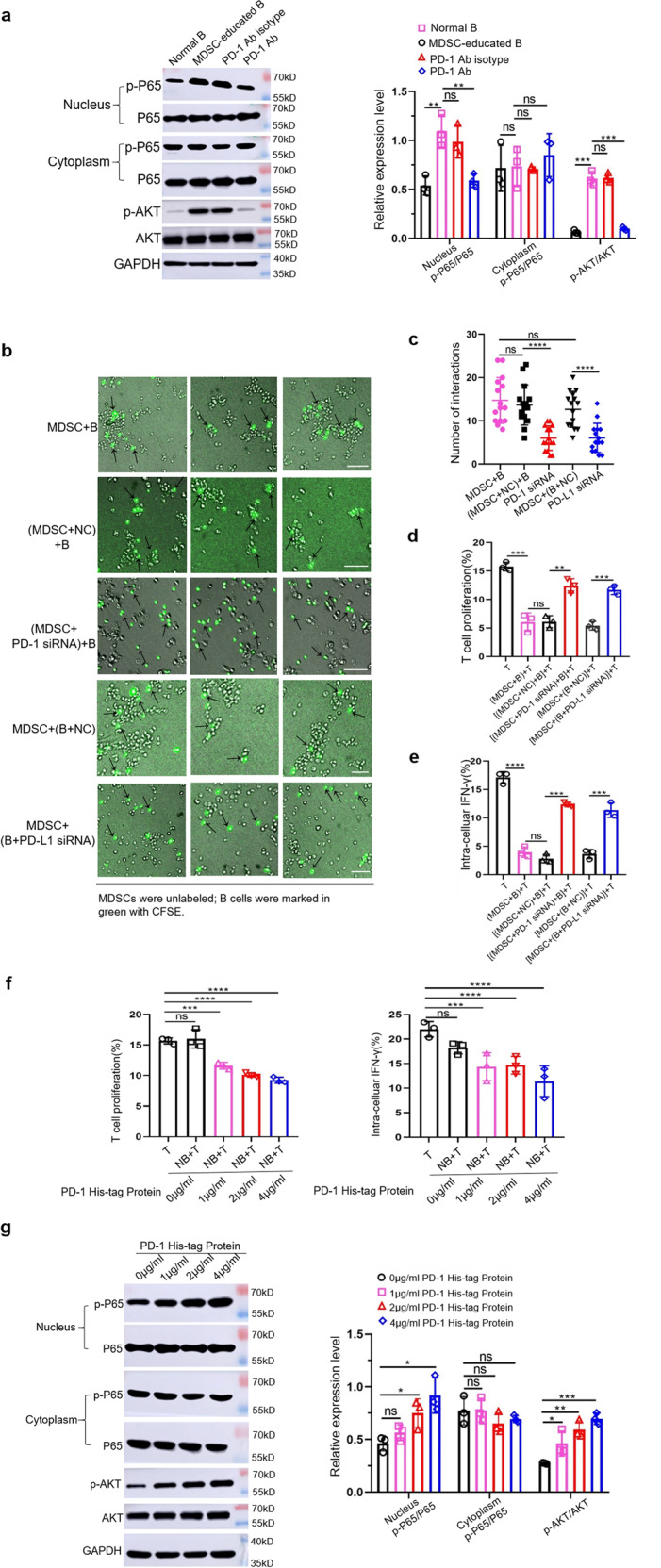
Tumor-derived MDSCs activate the PI3K/AKT/NF-κB signaling pathway in B cells through PD-1/PD-L1 axis. **a** Tumor-derived MDSCs were cocultured with normal B cells in the presence or absence of anti-PD-1 Ab and the protein levels of AKT, p-AKT, P65 and p-P65 were measured by western blot (*n* = 3). **b**, **c** MDSCs transfected with PD-1 siRNA, or B cells transfected with PD-L1 siRNA were cocultured with either B cells or MDSCs. For each group, we selected three different fields of view to analyze the mutual contacts between MDSCs with B cells by live cell imaging (*n* = 3). Scale bar: 50 µm. NC negative control. **d** B cells were isolated from the above five groups and co-incubated with T cells for 48 h. T cell proliferation and IFN-γ secretion (**e**) were measured (*n* = 3). **f** Normal B cells were stimulated with different concentrations of recombinant PD-1 His-tagged protein (R&D Systems, USA) and cultured with T cells for 48 h. T cell proliferation and IFN-γ secretion (*n* = 3), as well as protein levels of AKT, p-AKT, P65, and p-P65 were measured (**g**). (In all experiments, Bar graphs and plots represent or include mean ± SD, respectively. ns no statistically significant, *p < 0.05, **p < 0.01, ***p < 0.001, ****p < 0.0001).

To investigate whether MDSCs activate the PI3K/AKT/NF-κB signaling pathway in B cells through the PD-1/PD-L1 axis, we first detected the protein levels of PI3K/AKT**/**NF-κB signaling pathway components in MDSC-educated B cells after blocking with anti-PD-1 Ab. We found that the levels of phosphorylated AKT and P65 were significantly lower in the anti-PD-1 Ab groups than those of the isotype groups (Fig. 3a), which provides preliminary evidence for supporting our hypothesis. To further prove our hypothesis that PD-1 on MDSCs and PD-L1 on B cells bind to each other and trigger the activation of PI3K/AKT/NF-κB signaling pathway in MDSC-educated Bregs, we transfected PD-1 siRNA into MDSCs or PD-L1 siRNA into B cells and then co-incubated with either B cells or MDSCs. We analyzed the interactions between MDSCs and B cells via live cell imaging and evaluated the immunosuppressive function of PD-1^−^PD-L1^+^ Bregs after using siRNA. The results showed that the cell–cell contacts of MDSCs and B cells in the siRNA group were significantly reduced compared to control group (Fig. 3b, c and Supplementary Video). Moreover, the inhibitions of T cell proliferation (Fig. 3d) and IFN-γ secretion (Fig. 3e) by MDSC-educated Breg cells in siRNA group were also significantly attenuated.^+^

In addition, to further confirm this mechanism, we used a recombinant mouse PD-1 His-tag protein to activate normal B cells. We showed that the activated B cells were able to inhibit T cell proliferation and IFN-γ secretion (Fig. 3f) to a certain extent. We next examined the phosphorylation levels of AKT and P65 in B cells activated by different concentration recombinant mouse PD-1 His-tag proteins. We found that the phosphorylation levels of AKT and P65 in activated B cells were higher than those in normal B cells (Fig. 3g). Therefore, all the above data indicated that PD-1 on MDSCs and PD-L1 on B cells act through receptor/ligand interactions, thereby activating PI3K/AKT/NF-κB signaling pathway in MDSC-educated Bregs to exert the immunosuppressive functions on T cell immune response.

### MDSCs are capable of inducing PD-1^−^PD-L1^+^ B cells in vivo

**Fig. 4:**
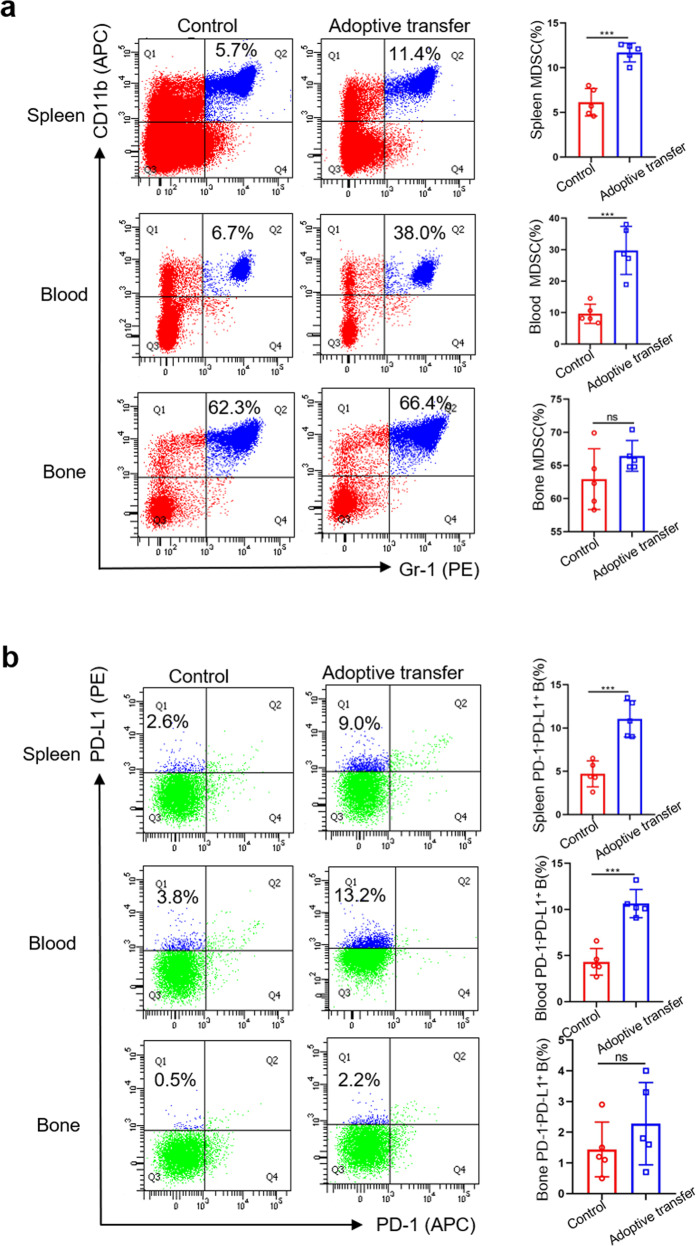
MDSCs are capable of inducing PD-1^−^PD-L1 B cells in vivo^+^. Normal mice were intravenously injected with congenic tumor-derived MDSCs (3 × 10^7^) every 2 days (*n* = 5). Normal mice were injected with PBS and used as the control group. After 1 week, the proportions of MDSCs (**a**) and PD-1^−^PD-L1^+^ B cells (**b**) in the spleen, peripheral blood, and bone marrow were measured by FC. (In all experiments, Bar graphs and plots represent or include mean ± SD, respectively. ns no statistically significant, ***p < 0.001).

To support the in vitro findings, we conducted an in vivo experiment to confirm that MDSCs can also educate PD-1^−^PD-L1^+^ Bregs in vivo. Congenic tumor-derived MDSCs were adoptively transferred to normal BALB/C mice three times a week, and the proportions of MDSCs and PD-1^−^PD-L1^+^ Bregs in the spleen, peripheral blood, and bone marrow were analyzed 1 week later. In the spleen and peripheral blood, the proportions of MDSCs and PD-1^−^PD-L1^+^ Bregs in the adoptive transfer group were higher than those in the control group (Fig. 4a, b), indicating that tumor-derived MDSCs could also stimulate PD-1^−^PD-L1^+^ Bregs with immunosuppressive functions in the peripheral blood or spleen in vivo. However, we observed no differences in the bone marrow (Fig. 4a, b). One possible reason is that MDSCs are generated in the bone marrow from common myeloid progenitor cells. They will migrate to peripheral lymphoid organs and tumors to exert different functional specialization but rarely homing to the bone marrow. Therefore, the function and fate of MDSCs depend on their localization. The development of MDSCs is governed by a complex network of signals and further evidences remain to be explored^12^.

### Treatment with anti-PD-1 mAbs and LY2940002 reduce tumorigenesis and the immunosuppressive effects of PD-1^−^PD-L1^+^ Bregs in vivo

**Fig. 5:**
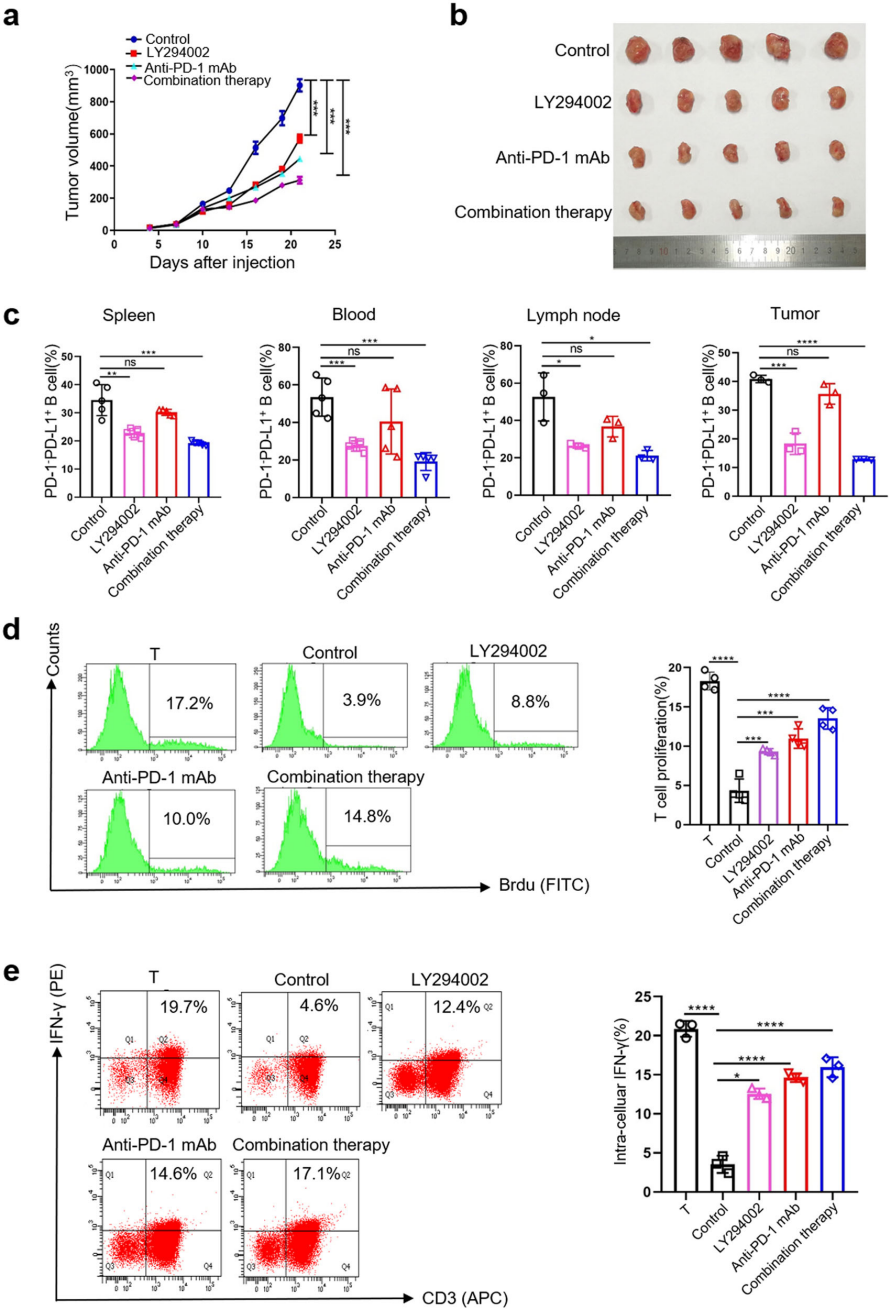
Treatment with anti-PD-1 mAbs and LY2940002 reduce tumorigenesis and the immunosuppressive effects of PD-1^−^PD-L1^+^ Bregs in vivo. **a**, **b** 4T1 tumor-bearing mice treated with anti-PD-1 mAbs or LY2940002 were euthanized at Day21 and the tumor volume were measured (*n* = 5). **c** Single-cell suspensions were prepared from the spleen, peripheral blood, LNs, and tumors in these 4T1 tumor-bearing mice, and the proportion of PD-1^−^PD-L1^+^ Bregs was measured by FC. **d**, **e** PD-1^−^PD-L1^+^ Bregs were sorted from the spleens of these 4T1 tumor-bearing mice and were cocultured with normal T cells for 48 h. The harvested cells were analyzed for T cell proliferation and IFN-γ secretion by FC. (In all experiments, Bar graphs and plots represent or include mean ± SD, respectively. ns no statistically significant, *p < 0.05, **p < 0.01, ***p < 0.001, ****p < 0.0001).

To support the in vitro findings, we conducted an in vivo experiment on mice with 4T1 tumors. Treatment of tumor-bearing mice with anti-PD-1 mAbs, LY294002, or combination therapy significantly inhibited tumor growth (Fig. 5a, b). The ratios of PD-1^−^PD-L1^+^ Bregs in the spleen, peripheral blood, LNs, and tumor tissues were reduced to a certain extent in the treatment group (Fig. 5c). Based on this result, we further sorted PD-1^−^PD-L1^+^ Bregs in the spleens of these tumor-bearing mice. Then, these PD-1^−^PD-L1^+^ Bregs were incubated with T cells to evaluate its immunosuppressive effects. Indeed, we found that PD-1^−^PD-L1^+^ Bregs in the anti-PD-1 mAb, LY294002 and combination therapy groups induce less inhibition of T cell proliferation (Fig. 5d) and IFN-γ secretion (Fig. 5e) than those of the control group. The efficacy of the combination therapy was better than that of individual treatment. In summary, these data suggested that MDSCs stimulation of B cells with immunosuppressive activity depends on the PD-L1/PI3K/AKT signaling pathway in vivo^+^.

### PD-1^−^PD-L1^+^ Bregs and MDSCs are colocalized in tumor tissues and associated with poor prognosis in breast cancer

**Fig. 6:**
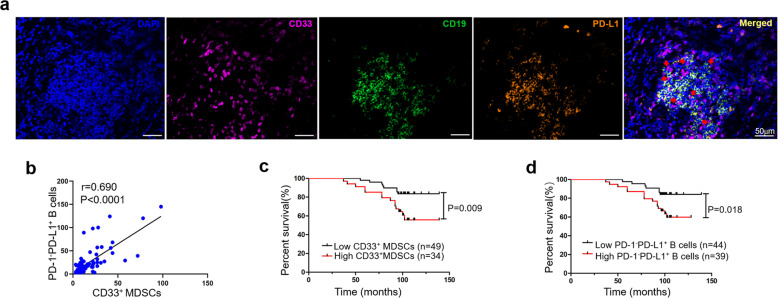
PD-1^−^PD-L1^+^ Bregs and MDSCs are colocalized in tumor tissues and associated with poor prognosis in breast cancer^+^. **a** Multiplex fluorescent immunohistochemistry staining of breast cancer tissue showing CD33 (purple), CD19 (green), and PD-L1 (orange) in close proximity (red arrows). Original images were taken at ×20 magnification. Scale bar: 50 µm. **b** The number of PD-1^−^PD-L1^+^ Bregs (*n* = 83) was correlated with the number of CD33^+^ MDSCs (*n* = 83). Kaplan–Meier analysis graph showing that MDSCs (**c**) and PD-1^−^PD-L1^+^ Bregs (**d**) were associated with the overall survival of patients (*n* = 83) with breast cancer.

If MDSCs can educate B cells to differentiate into PD-1^−^PD-L1^+^ Bregs in tumor tissue in a manner that depends on cell–cell interactions, these cells would be expected to be located in close proximity to B cells in tumor tissue samples of patients. To assess this, we used tumor tissue samples from 83 breast cancer patients and labeled MDSCs and B cells by multiplex fluorescent immunohistochemistry. The colocalization of CD33^+^ MDSCs and PD-1^−^PD-L1^+^ Bregs was observed in 43 (51.8%) tumor tissue samples (Fig. 6a), and the degree of colocalization was listed in Supplementary Table 1. Accordingly, we evaluated the correlation between CD33^+^ MDSCs and PD-1^−^PD-L1^+^ Bregs and observed an obvious positive correlation (Fig. 6b), indicating that MDSCs may educate PD-1^−^PD-L1^+^ Bregs to exert immunosuppressive effects in tumor tissues.

Studies have shown that the level of MDSCs is associated with the prognosis of patients^31,32^. Consistent with these findings, Kaplan–Meier analyses indicated that high levels of MDSCs (>12 median level) were associated with worse overall survival than low levels of MDSCs (≤12) (Fig. 6c). As shown in Table 1, the correlation between CD33^+^ MDSCs and the clinicopathological characteristics of patients, including age, LN metastasis, TNM stage, progesterone receptor (PR), and human epidermal growth factor receptor 2 (HER2), was not statistically significant, except for estrogen receptor (ER). We next analyzed the relationship between the number of PD-1^−^PD-L1^+^ Bregs and patient survival. Comparing patients with high (>15 median level) versus low (≤15) numbers of PD-1^−^PD-L1^+^ Bregs, the overall survival rates were significantly lower for those in the group with higher numbers of PD-1^−^PD-L1^+^ Bregs (Fig. 6d). Further analysis of the relationships between the number of PD-1^−^PD-L1^+^ Bregs and the clinicopathological features of patients was performed. There was no statistical significance associated with age, LN metastasis, TNM stage, ER, PR, or HER2 (Table 1). Moreover, as shown in Table 2, univariable analysis found that the level of MDSCs and PD-1^−^PD-L1^+^ Bregs, TNM stage, LN metastasis were high-risk factors related to overall survival, while ER showed a protective effect. And multivariable Cox regression analysis showed that the level of MDSCs and LN metastasis were independent high-risk factors (Table 2). These findings indicated that MDSCs and PD-1^−^PD-L1^+^ Bregs play immunosuppressive roles, are associated with poor prognosis in breast cancer and the high levels of MDSCs are identified as an independent prognostic marker for shorter survival of patients with breast cancer.^+^

**Table 1:** The relationship between CD33^+^ MDSCs, PD-1^−^PD-L1^+^ B cells, and clinicopathologic features of the patients.

Variables	All cases	CD33^+^ MDSCs		*P* value	PD-1^−^PD-L1^+^CD19^+^ B cells		*P* value
		Low	High		Low	High	
Age							
<50 years	42	21 (50.0%)	21 (50.0%)	0.090	18 (42.9%)	24 (57.1%)	0.061
≥50 years	41	28 (68.3%)	13 (31.7%)		26 (63.4%)	15 (36.6%)	
LN metastasis							
Yes	41	25 (61.0%)	16 (39.0%)	0.723	23 (56.1%)	18 (43.9%)	0.578
No	42	24 (57.1%)	18 (42.9%)		21 (50%)	21 (50%)	
TNM stage							
I	27	16 (59.3%)	11 (40.7%)	0.875	15 (55.6%)	12 (44.4%)	0.536
II	41	25 (61.0%)	16 (39.0%)		23 (56.1%)	18 (43.9%)	
III	15	8 (53.3%)	7 (46.7%)		6 (40%)	9 (60%)	
ER							
Positive	56	39 (69.6%)	17 (30.4%)	0.005	29 (51.8%)	27 (48.2%)	0.747
Negative	27	10 (37.0%)	17 (63.0%)		15 (55.6%)	12 (44.4%)	
PR							
Positive	46	31 (67.4%)	15 (32.6%)	0.084	26 (56.5%)	20 (43.5%)	0.475
Negative	37	18 (48.6%)	19 (51.4%)		18 (48.6%)	19 (51.4%)	
HER2							
Positive	42	26 (61.9%)	16 (38.1%)	0.591	21 (50%)	21 (50%)	0.578
Negative	41	23 (56.1%)	18 (43.9%)		23 (56.1%)	18 (43.9%)

**Table 2:** Cox proportional hazards models for overall survival of patients with breast cancer.

Variables	Univariable		Multivariable	
	HR (95% CI)	*P* value	HR (95% CI)	*P* value
CD33^+^ MDSCs	2.944 (1.254–7.149)	0.013	3.175 (1.327–7.599)	0.009
PD-1^−^PD-L1^+^ B cells	2.800 (1.141–6.871)	0.025		
Age (≥50 years vs. <50 years)	0.780 (0.337–1.806)	0.562		
LN metastasis(Yes vs. No)	2.442 (0.995–5.995)	0.051	2.620 (1.065–6.447)	0.036
TNM stage (III + II vs. I)	3.461 (1.024–11.700)	0.046		
ER (positive vs. negative)	0.428 (0.185–0.988)	0.047		
PR (positive vs. negative)	0.630 (0.272–1.458)	0.280		
HER2 (positive vs. negative)	1.526 (0.652–3.572)	0.330		

## Materials and methods

### Animals and cell lines

Female BALB/C mice aged 6–8 weeks were purchased from Sibeifu Corporation (Beijing, China). All mice were maintained in a pathogen-free environment at Tianjin Medical University Cancer Institute and Hospital. Animal care followed the guidance of the Institutional Animal Care and Use Committee. 4T1 mammary cells were obtained from the American Type Culture Collection, authenticated and tested for mycoplasma contamination. For tumor implantation, BALB/C mice were shaved on the right side of the groin and subcutaneously injected with 10^6^ 4T1 tumor cells. After 21 days, the mice that had been successfully inoculated with tumors were used for experiments. All experiments were approved by the Ethics Committee for Animal Experiments at the Tianjin Medical University Cancer Hospital and Institute.

### Cell isolation

According to the manufacturer’s instructions, B cells or T cells were isolated from the spleens of normal mice by negative selection using a mouse B cell or T cell isolation kit (>93% purity, Miltenyi Biotec, Germany), respectively. MDSCs were purified from the spleens of tumor-bearing mice by using Gr-1 microbeads (>95% purity, Miltenyi Biotec). MDSC-educated B cells were positively sorted from the MDSC and B cell coincubation system using CD19 magnetic beads (>95% purity, Miltenyi Biotec). PD1^−^PD-L1^+^ B cells were isolated from the spleens of tumor-bearing mice by a fluorescence-activated cell sorter (>95% purity, FACS, BD Biosciences, San Jose, CA, USA). All sorted cells were cultured in RPMI 1640 medium with 10% fetal bovine serum.

### In vitro coculture experiments

#### Coculture of MDSCs and B cells

Normal B cells were cocultured with tumor-derived MDSCs at a ratio of 1:5 in six-well flat-bottom plates in the presence of lipopolysaccharide (LPS, 25 µg/ml, Sigma, USA) and anti-mouse CD40 (10 μg/ml, Biolegend, USA) for 24 h.

#### Coculture of B cells and T cells

Normal T cells were cocultured with B cells at a ratio of 1:1 and were stimulated with anti-CD3/CD28 beads (15 µg/ml, Gibco, Grand Island, NY, USA) for 48 h.

#### Cell proliferation assays

Bromodeoxyuridine (BrdU) cell proliferation assay kit (BD Biosciences) was used to detect the proliferation of cultured cells. Cells (10^6^) were stimulated with BrdU (3 μg/ml) for 12 h and then stained with surface antigens. Harvested cells were fixed and permeabilized with BD Cytofix/Cytoperm Buffer. Next, the cells were incubated with BD Cytoperm Permeabilization Buffer Plus. Then the cells were re-fixed and treated with DNase to expose incorporated BrdU. Finally, the cells were stained with BrdU and intracellular antigens to detect the proliferation activity.

#### Flow cytometry (FC)

Single-cell suspensions from mouse tissue or cultured cells were used for the detection of cell surface molecules. The following Abs were used for FC staining: PE-Gr-1, APC-CD11b, PerCP-CD19, FITC-PD-1, APC-PD-1, PE/Cy7-PD-L1, PE-PD-L1, APC-CD3, and isotype controls (Catalog numbers were listed in Supplementary Table 2). Cells were incubated with the Abs for 30 min in dark at 4 °C and washed twice with PBS before FC analysis.

For intracellular cytokine staining, cells were stimulated with PMA (50 ng/ml, Sigma-Aldrich), ionomycin (1 μl/ml, Sigma-Aldrich) and GolgiStop (0.67 μl/ml, BD Biosciences) for 4 h. Then the cells were fixed with fixation/permeabilization solution (BD Biosciences) and incubated with PE-IFN-γ intracellular staining Abs (Biolegend) for 25 min. After that, cells were resuspended in 200 μl of cell staining buffer for FC analysis.

#### Live cell imaging

PD-1 or PD-L1 siRNA (GenePharma, Shanghai, China) with their control siRNA were transfected into tumor-derived MDSCs or normal B cells, respectively. B cells labeled with carboxyfluorescein diacetate succinimidyl ester (CFSE, 5 mM) were cocultured with MDSCs, stimulated with LPS and anti-mouse CD40, then placed directly into a 15 mm glass bottom dish to be recorded by live cell imaging for 24 h. For the quantitative data, the interactions between MDSCs and B cells were quantified under LEICA CTR6000 microscopy at ×20 magnification by two independent professionals. Five fields of view were randomly selected for evaluating the number of the interactions between MDSCs and B cells.

#### Western blotting

Cell were lysed in lysis buffer (2% SDS, 10% glycerol, 10 mM Tris, pH 6.8, 100 mM DTT), boiled for 10 min and then were separated by SDS-PAGE and transferred to polyvinylidene difluoride membranes. The membranes were blocked with 5% milk for 1 h and incubated with the following Abs (Catalog numbers were listed in Supplementary Table 2) overnight at 4 °C: GAPDH (1:3000), ERK (1:1000), PLCγ2 (1:1000), AKT (1:1000), P65 (1:1000), p-ERK (1:1000), p-PLCγ2 (1:1000), p-AKT (1:1000) and p-P65 (1:1000). Then, the membranes were incubated with horseradish peroxidase-conjugated anti-rabbit or anti-mouse IgG Ab for 1.5 h and imaged using Image Studio software for analysis.

#### Nuclear extraction

Nuclear Extraction Kit (Abcam) was used to detect the NF-κB p65 phosphorylation levels. A total of 10^7^ harvested cells were washed with PBS and resuspended in 1 mL of 1× Pre-Extraction Buffer, incubated on ice for 10 min, and then vortexed vigorously for 10 s and centrifuged for 1 min at 12,000 rpm. The cytoplasmic extracts (supernatant) were carefully removed and quantified for application. The nuclear extracts (pellet) were resuspended in 200 µL Extraction Buffer (with DTT and PIC) and incubated on ice for 15 min. The mix was centrifuged for 10 min at 14,000 rpm at 4 °C and the supernatant was transferred into a new tube for application.

#### In vivo experiments

For adoptive transfer experiments, normal BALB/C mice were intravenously injected with congenic tumor-derived MDSCs (3 × 10^7^) every 2 days. A week later, single-cell suspensions were prepared from the spleen, blood, and bone of these mice to determine the percentages of MDSCs and PD-1^−^PD-L1^+^ B cells.

For drug treatment experiments, BALB/C mice were shaved on the right side of the groin and subcutaneously injected with 10^6^ 4T1 tumor cells. When the tumor size was large enough to touch and measure (~1 week), LY294002 (25 mg/kg, MedChemExpress, USA), anti-PD-1 mAb (10 mg/kg, Pembrolizumab, MedChemExpress, USA), or PBS were administered via intraperitoneal injection twice a week. Tumor diameters were monitored three times a week using calipers, and tumor volume (mm^3^) was calculated with the following formula: tumor volume = (longest axis × shortest axis × shortest axis)/2. After 3 weeks, all mice were euthanized. The percentages of PD-1^−^PD-L1^+^ Bregs in the spleen, blood, LNs, and tumor were determined by FC. Meanwhile, PD-1^−^PD-L1^+^ Bregs sorted from spleen were cocultured with normal T cells to evaluate their immunosuppressive activity. For animal studies, mice were randomly assigned to different groups according to the random number table and the investigators were blinded to the group assignment during the process of experiment and analysis.^+^

#### Multiplex fluorescent immunohistochemistry and multispectral imaging

Based on the preliminary result, we calculated sample size on “power and sample size” website (http://powerandsamplesize.com/). The result showed that if we collect 71 samples, we can achieve a power of 0.8 and type I error rate of 5%. All breast cancer tumor samples were obtained from Tianjin Medical University Cancer Hospital. The study was approved by the Ethics Committee of Tianjin Cancer Institute and Hospital and obtained from informed consent by all subjects. None of the patients had received chemotherapy or radiotherapy before surgery. Patients with infectious diseases, autoimmune disease or multiple primary cancers were excluded. All patients were followed for more than 100 months.

The paraffin-embedded slides were stained with multiplex fluorescence using the PerkinElmer Opal 7-Color technology Kit (NEL81001KT, PerkinElmer, USA) containing seven fluorophores. Slides were deparaffinized successively in xylene and rehydrated in ethanol. Antigen retrieval was performed in EDTA (pH = 9.0) with microwave treatment. Then the samples were blocked with an Ab-blocking buffer for 10 min at room temperature and incubated overnight with primary antibodies overnight at 4 °C. Next day, the secondary antibodies (polymer HRP Ms/Rb) were incubated for 10 min at room temperature. TSA Visualization and signal amplification were performed by Opal TSA plus. Then the Ab-TSA complex was removed by heating with EDTA buffer in microwave. Each multiplex staining was repeated by these staining steps in series. At last, 4′,6-diamidino-2-phenylindole (DAPI) was used to stain the cell nuclei. Imaging was performed using the Mantra quantitative pathology imaging system (PerkinElmer). Manual counting was performed in five random fields at ×20 magnification and the mean numbers of all fields from each patient sample were calculated. The pathologists were blinded to the group assignment when evaluating the results.

### Statistical analysis

The results are presented as mean ± SD of at least three experiments, and *t* test or one-way ANOVA analysis of variance was employed for analyzing the difference between groups differences by Prism v8.0 (GraphPad Software). The *χ*^2^ test was used to assess the relationship of clinicopathologic features of patients and CD33^+^ MDSCs, PD-1^−^PD-L1^+^ Bregs. The correlations between CD33^+^ MDSCs and PD-1^−^PD-L1^+^ Bregs were estimated by Spearman correlation analysis. Kaplan–Meier survival analysis was performed using ‘low’ or ‘high’ classifications according to the median of the number of PD-1^−^PD-L1^+^ Bregs and MDSCs. Cox regression proportional hazard models were used to quantify hazard ratios for death from breast cancer in both univariable and multivariable analysis, adjusted for age, LN metastasis, TNM stage, ER, PR or HER2. A two-tailed *P* value less than 0.05 was considered statistically significant.^+^

## Discussion

**Fig. 7:**
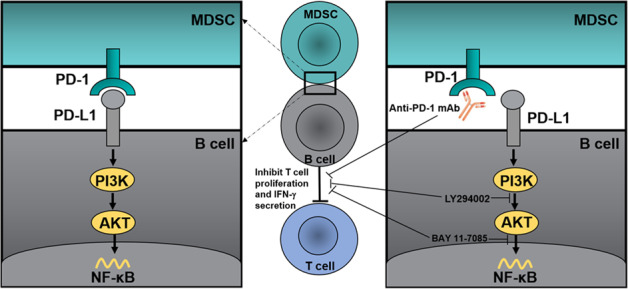
Schematic representation of the MDSCs regulation on B cells in breast cancer. Breast cancer-derived MDSCs regulate the immunosuppressive functions of PD-1^−^PD-L1^+^ B cells through PD-L1/PI3K/AKT/NF-κB axis. Anti-PD-1 Abs, LY294002, and BAY 11-7085 can reverse MDSC-educated B cell-mediated inhibition of the T cell immune response^+^.

The underlying mechanisms involved in the influence of MDSCs on B cells in cancer remain largely unknown. In our previous study^25^, we showed that MDSCs educate a unique Breg subset of PD-1^−^PD-L1^+^ B cells that exert a robust inhibitory effect on the T cell immune response. Here, we revealed a new mechanism by which MDSCs regulate the immunosuppressive functions of PD-1^−^PD-L1^+^ Bregs in breast cancer (Fig. 7). We showed that MDSCs are capable of inducing PD-1^−^PD-L1^+^ B cells in vivo. Most importantly, PD-1^−^PD-L1^+^ Bregs were associated with poor prognoses of patients with breast cancer. These findings contribute to a more complete understanding of the negative regulatory functions of MDSCs on the multicellular network of immune responses and have important implications for the differentiation of B cells and the formation of Bregs.^+^

The binding of PD-1 and PD-L1 exists in different types of immune cells, such as neutrophils and T cells^33^, macrophages and T cells^34^, and MDSCs and T cells^35^. In 4T1 tumor-bearing mice, increased expression of PD-1/PD-L1 axis components on B cells and MDSCs^25^ suggests the possible engagement of this pathway between MDSCs and B cells, inducing immune effects during tumor progression. Our findings demonstrated that blocking the PD-1 and PD-L1 interaction between MDSCs and B cells can reverse the inhibition of T cell proliferation and IFN-γ secretion by MDSC-educated B cells, indicating that the PD-1/PD-L1 axis may play a decisive role in the relationship between MDSCs and B cells.

The PD-1/PD-L1 axis is a key determinant of immune homeostasis in infection, tolerance, and cancer^36^. We next investigated the PD-1/PD-L1-related signaling pathway in normal and PD-1^−^PD-L1^+^ B cells^37^. Our results showed activation of the AKT pathway in PD-1^−^PD-L1^+^ B cells. Moreover, inhibiting the upregulation of the AKT pathway could also reverse the PD-1^−^PD-L1^+^ Breg-mediated inhibition of T cell proliferation and IFN-γ secretion. It is well known that the PI3K/AKT signaling pathway is essential for B cell development and differentiation^27,38,39^. In the present work, we expanded previous observations and identified the role of this pathway in the differentiation of Bregs, or, more precisely, the PD-1/PD-L1 axis and AKT pathway are required for MDSC-mediated effects on B cells. Liu et al.^40^ found that endoplasmic reticulum stress leads to increased levels of exosomal miR-23a-3p derived from hepatoma cells, and miR-23a-3p upregulated the expression of PD-L1 on macrophages by regulating the AKT pathway. This seems to contradict our findings, but it may not be contradictory. Although the role of PD-1 is well understood, a potential “reverse signal” through PD-L1 has rarely been explored^41^. Some evidence has shown that recombinant PD-1 stimulates PD-L1 in tumor cells, resulting in antiapoptotic signaling^42^. The present study is the first demonstration that stimulation of PD-L1 by recombinant PD-1 induces B cells to exert an immunosuppressive effect on the T cell immune response. Furthermore, anti-PD-1 Abs significantly reduced the activity of the PI3K/AKT pathway in PD-1^−^PD-L1^+^ Bregs. Thus, these results suggest that the PI3K/AKT pathway regulates the expression of PD-L1, whereas the PD-1/PD-L1 axis also regulates the PI3K/AKT signaling pathway.

Based on the in vitro results, it could be hypothesized that MDSCs can stimulate PD-1^−^PD-L1^+^ Bregs in vivo to expand their negative effects. Wang et al.^43^ showed that B cell differentiation in tumor-bearing mice was impaired, but the differentiation of immunosuppressive Bregs was increased, and adoptive transfer of MDSCs reduced B cell subsets, which were consistent with our results. We identified that adoptive transfer of MDSCs increased immunosuppressive subset of PD-1^−^PD-L1^+^ Bregs in the peripheral blood and spleen in vivo. We also found a significant positive correlation between MDSCs and PD-1^−^PD-L1^+^ Bregs in tumor tissue from breast cancer patients. Furthermore, the colocalization of MDSCs and B cells in tumor tissue also supported the interaction of these two cells by cell–cell contact.^+^

We further verified the mechanism in vivo and found that treatment of tumor-bearing mice with anti-PD-1 Abs reduced the immunosuppressive effects of PD-1^−^PD-L1^+^ B cells. LY294002 not only weakened the immunosuppressive effects of PD-1^−^PD-L1^+^ B cells but also reduced the proportion of PD-1^−^PD-L1^+^ B cells in vivo. It is consistent with the in vitro results. A previous study reported that a selective PI3K inhibitor enhanced the efficacy of anti-PD-L1 Abs through partially abrogating local immunosuppression mediated by MDSCs^44^. What we revealed here is that PI3K inhibitor can reverse the effect of MDSC-mediated B cell regulation. In addition, combination therapy of PI3K inhibitor and anti-PD-1 Ab showed a better effect than individual treatments, which may provide a new mechanism for combined immunotherapy.^+^

The associations between B lymphocytes and patient prognosis have been identified in some studies^45–47^. However, the potential mechanism by which the different subsets exert clinical and biological effects in the tumor has not been clarified. Guan et al.^48^ observed that CD19^+^ B cells were coincident with PD-L1 in invasive breast carcinoma. Consistent with this finding, PD-1^−^PD-L1^+^ Bregs were found in many tumor samples and correlated with poor prognosis in breast cancer. In the present work, we observed a significant positive correlation between CD33^+^ MDSCs and PD-1^−^PD-L1^+^ Bregs, and the level of MDSCs was an independent prognostic factor and might be a promising biomarker for evaluating breast cancer patient prognosis. Bergenfelz et al.^49^ reported that the high level of MDSCs was significantly correlated with ER-negative breast cancer, and the higher inflammatory activity in ER-negative breast cancer may consequently promote the accumulation of MDSCs^50,51^. This is in accordance with our results that the level of MDSCs was relatively lower in ER-positive breast cancer.

Taken together, our results suggest a new regulatory mechanism by which MDSCs stimulate PD-1^−^PD-L1^+^ Breg-mediated immunosuppression in human tumors (Fig. 7). Tumor-derived MDSCs activate the PI3K/AKT/NF-κB pathway in B cells by a direct receptor-ligand interaction, and MDSC-educated PD-1^−^PD-L1^+^ Bregs suppress the T cell immune response, thereby contributing to tumor progression. Accordingly, immunotherapies that interfere with the PD-1/PD-L1 axis and activation of the PI3K/AKT/NF-κB pathway can restore immune dysfunction in tumors, which further emphasizes the role of the PD-1/PD-L1 axis in tumor pathogenesis. This study is also the first demonstration that the intracellular biochemistry of this “back” signaling of PD-L1 that can function as a receptor to “back” transmit signals into B cells and affect their functions. In addition, we suggest the prognostic importance of tumor-infiltrating B cells in patients with breast cancer. Further study of the dynamic changes and the localization of PD-1^−^PD-L1^+^ B cell immunomodulatory effects may help us better understand their roles in tumor progression, contributing to a novel strategy for immunotherapy.

## Supplementary information

Supplementary Figure 1

Supplementary Figure 2

Supplementary Figure 3

supplementary Table 1

supplementary Table 2

Supplementary Figure legend

Supplementary Video

Supplementary Video

Supplementary Video

Supplementary Video

Supplementary Video
