# Supplementary figures and images for: Myeloid-derived suppressor cells regulate the immunosuppressive functions of PD-1−PD-L1+ Bregs through PD-L1/PI3K/AKT/NF-κB axis in breast cancer
Source: Cell Death Dis. 2021 May 9;12(5):465. doi: 10.1038/s41419-021-03745-1 (PMC8107179; doi:10.1038/s41419-021-03745-1)

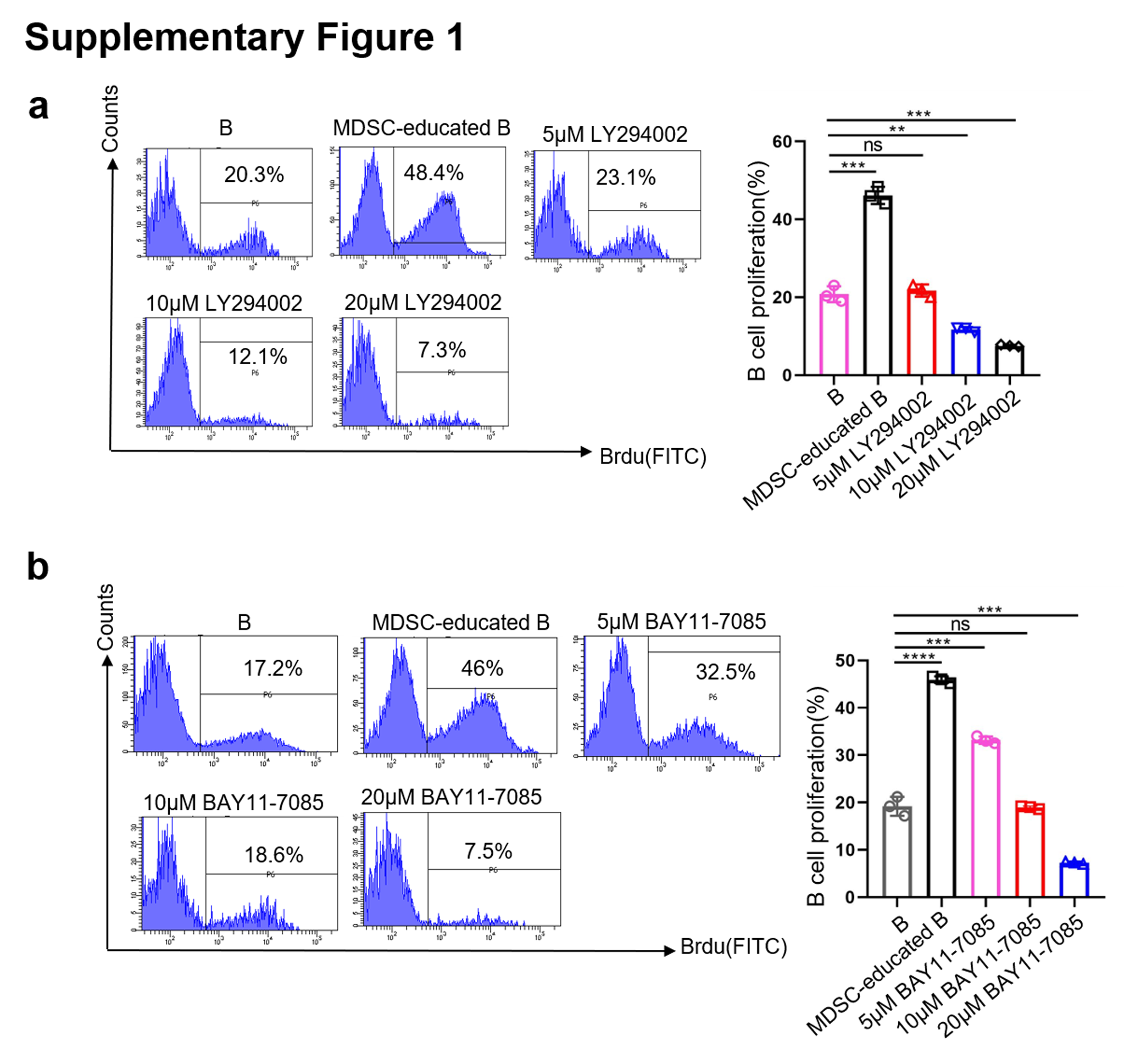

Supplement: Supplementary file 1 — Supplementary Figure 1 [file 41419_2021_3745_MOESM1_ESM.tif]

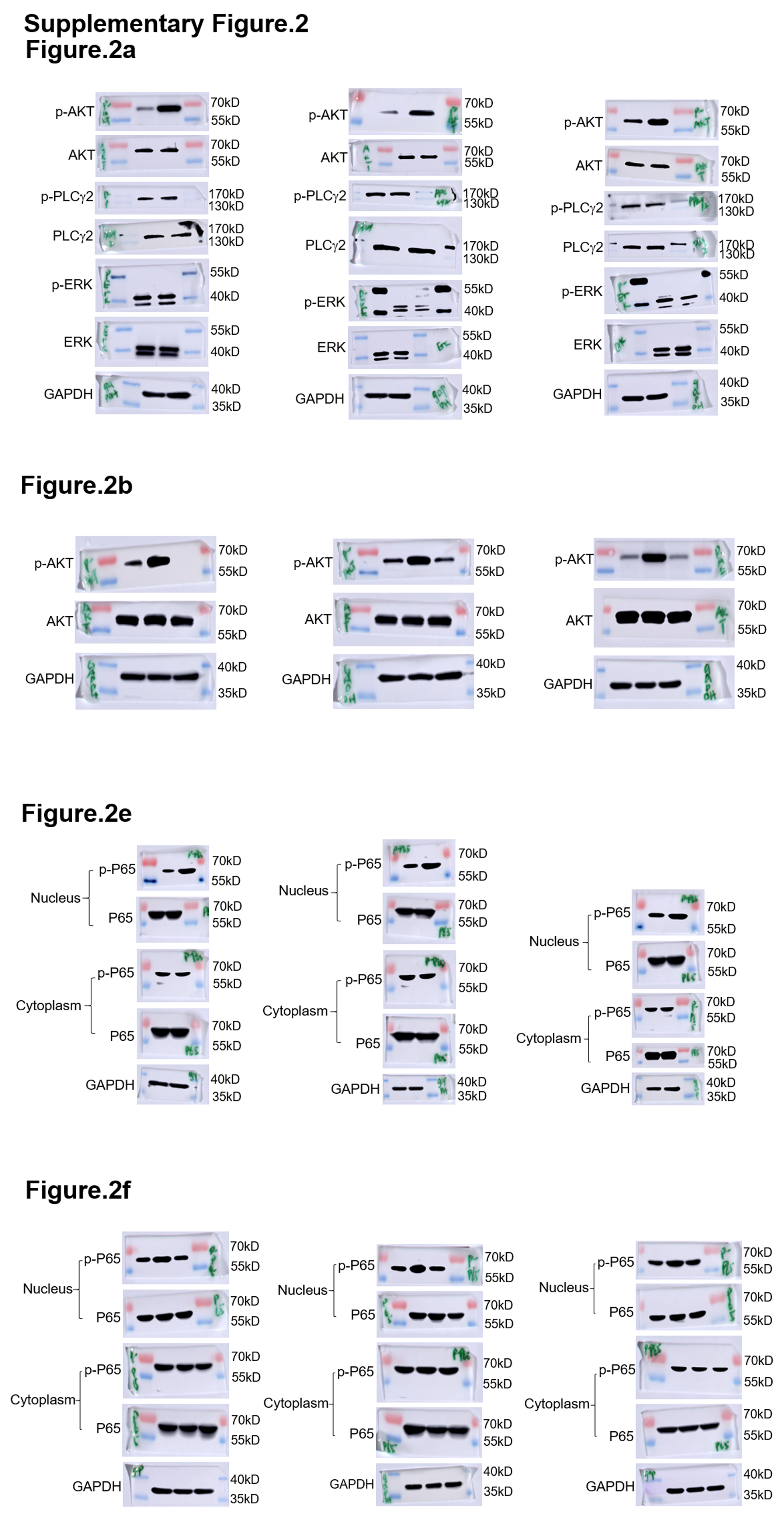

Supplement: Supplementary file 2 — Supplementary Figure 2 [file 41419_2021_3745_MOESM2_ESM.tif]

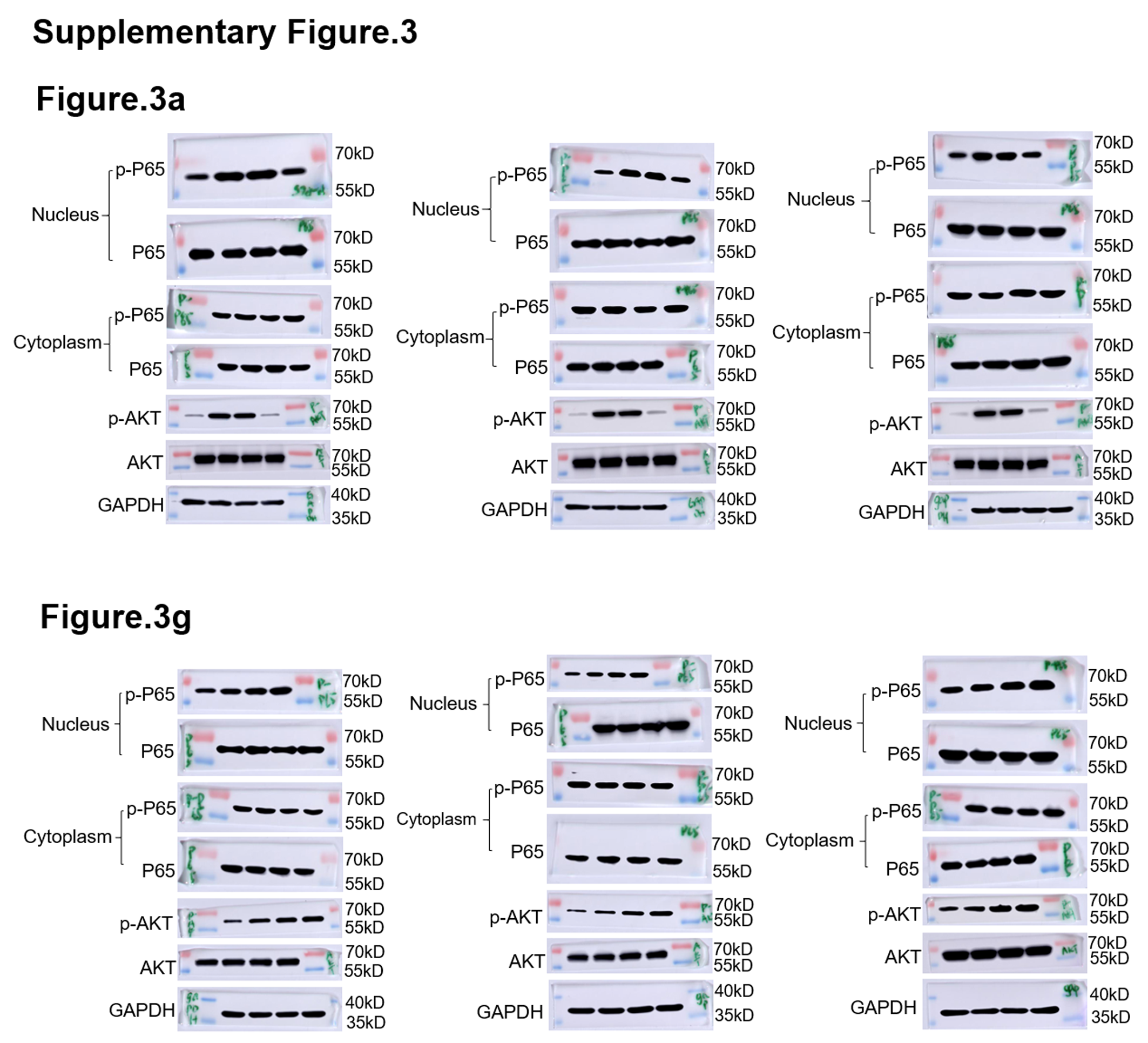

Supplement: Supplementary file 3 — Supplementary Figure 3 [file 41419_2021_3745_MOESM3_ESM.tif]
