# Supplementary material for: Myeloid-derived suppressor cells regulate the immunosuppressive functions of PD-1−PD-L1+ Bregs through PD-L1/PI3K/AKT/NF-κB axis in breast cancer
Source: Cell Death Dis. 2021 May 9;12(5):465. doi: 10.1038/s41419-021-03745-1 (PMC8107179; doi:10.1038/s41419-021-03745-1)
Supplement: Supplementary file 6 — Supplementary Figure legend [file 41419_2021_3745_MOESM6_ESM.docx]

**Supplementary Figure.1 The effects of different concentration of LY294002 or BAY 11-7085 on B cells.** MDSC-educated B cells were treated with or without different concentration of LY294002 (a) or BAY 11-7085 (b). Normal B cells incubated alone served as a control group. And the cells were harvested to detect the proliferation activity of B cells by BrdU (n=3). (In all experiments, Bar graphs and plots represent or include mean ± SD, respectively. ns: no statistically significant, **p<0.01, ****p<0.0001).

**Supplementary Figure.2** All original triplicates of all western blots in Figure 2.

**Supplementary Figure.3** All original triplicates of all western blots in Figure 3.

**Supplementary Video. The mutual contacts between MDSCs with B cells.** MDSCs transfected with PD-1 siRNA, or B cells transfected with PD-L1 siRNA were cocultured with either B cells or MDSCs. For each group, we recorded the mutual contacts between MDSCs with B cells by live cell imaging for 24h. MDSCs were unlabeled and B cells are labeled green with CFSE. NC: negative control.
